# C-Phycocyanin exerts anti-cancer effects via the MAPK signaling pathway in MDA-MB-231 cells

**DOI:** 10.1186/s12935-018-0511-5

**Published:** 2018-01-25

**Authors:** Liangqian Jiang, Yujuan Wang, Guoxiang Liu, Huihui Liu, Feng Zhu, Huanhuan Ji, Bing Li

**Affiliations:** Department of Genetics and Cell Biology, Basic Medical College, Qingdao University, 308 Ningxia Road, Qingdao, 266071 China

**Keywords:** C-Phycocyanin, Cell cycle arrest, Apoptosis, COX-2, MAPK, MDA-MB-231 cells

## Abstract

**Background:**

Triple-negative breast cancer is a biological subtype of breast cancer, which is unresponsive to conventional chemotherapies and has a poor prognosis. C-Phycocyanin (C-PC), a marine natural purified from *Spirulina platensis*, has been investigated that has anti-cancer function. The mitogen activated protein kinase (MAPK) pathway plays a crucial role in the development and progression of cancer. Therefore, we would like to study the anti-cancer effects of C-phycocyanin in the treatment of triple-negative breast cancer, and explore the role of MAPK pathway in the anti-tumor effects of C-phycocyanin.

**Methods:**

Cell proliferation, cell cycle, cell apoptosis and cell migration were explored in breast cancer MDA-MB-231 cell lines. AKT, MAPK and membrane death receptor signaling were evaluated in MDA-MB-231 cell lines.

**Results:**

Our study indicated that C-phycocyanin inhibited cell proliferation and reduced colony formation ability of MDA-MB-231 cells. Furthermore, C-phycocyanin induced cell cycle G0/G1 arrest by decreasing protein expression levels of Cyclin D1 and CDK-2 and increasing protein expression levels of p21 and p27. In addition, C-phycocyanin induced cell apoptotic by activating cell membrane surface death receptor pathway. Besides, C-phycocyanin down-regulated the protein expression levels of cyclooxygenase-2, and further inhibited MDA-MB-231 cells migration. We also found cell death induced by C-phycocyanin was carried through the MAPK signaling pathways. C-Phycocyanin was able to induce MDA-MB-231 cell apoptosis by activating p38 MAPK and JNK signaling pathways while inhibiting ERK pathway.

**Conclusions:**

C-Phycocyanin exerted anti-cancer activity via the MAPK signaling pathway in MDA-MB-231 cells.

## Introduction

The most common diagnosed cancer for women is breast cancer, meanwhile, it also has a high mortality rate in cancer worldwide for women. In the world, it was an estimated 1.7 million cases of breast cancer, and the number of deaths was 521,900 cases in 2012. It is serious that breast cancer alone holds 25% of all female cancer cases and 15% of female cancer cases die from breast cancer in 2012 [1]. It is expected that breast cancer will account for 30% of all new diagnosed cancer and the new mortality rate of breast cancer will hold 14% of all diagnosed cancer among females in 2017 [2]. The triple-negative breast cancer (TNBC) is a common biological subtype of breast cancer and the pathologic features are the progesterone receptor (PR)-negative, the estrogen receptor (ER)-negative and the HER2-negative [3, 4]. The cancer progression of TNBC patients usually contains higher early recurrence rates, poorer disease-specific survival, more aggressive process, and higher visceral and central nervous system metastases [4]. Although clinical physicians currently lack treatment strategies for TNBC patients, the discovery of new drugs and the development of selective therapy provide great hope for the future.

Recently, more and more researches report that marine natural products have promising anticancer activity, meanwhile, with little or no side effects [5]. C-Phycocyanin (C-PC), a marine protein purified from *Spirulina platensis* is, has been confirmed that has the function of inhibiting tumorigenesis. A wide range of pharmacological studies have confirmed that C-phycocyanin has many functions, such as anti-cancer [6], antioxidant [7], anti-inflammatory activity [7] and light-induced cytotoxicity [8] and many more. More studies have shown that C-phycocyanin exerts anti-cancer effect in various cancer cell types (such as lung cancer [9], liver cancer [6], colon cancer [10], breast cancer [8] and Leukemia [11] and so on) in vitro and in vivo.

It is widely accepted that The mitogen activated protein kinase (MAPK) pathway plays a key role in the development and progression of cancer [12], which include cell proliferation, senescence, differentiation, migration, apoptosis and many more [13–15]. There are three major MAPK cascades in humans: c-Jun *N*-terminal kinase (JNK), extracellular signal-regulated kinase (ERK1/2) and p38 MAPK. JNK can function as a pro-apoptotic kinase in response to a variety of extracellular stimuli, including chemotherapeutic drugs, tumor necrosis factor (TNF), UV irradiation and cytokines. Some studies had proved that the JNK pathway activates caspases and regulates apoptosis-related proteins, including Bcl-2 and Bax [16]. The ERK activation is associated with the pathogenesis, progression, and oncogenic behavior of human breast cancer and colorectal cancer [17, 18]. The effect of p38 MAPK signaling is diverse, and p38 MAPK has been shown to promote cell death or enhance cell growth and survival [19, 20]. Thus, the MAPK pathway is one important signaling pathway associated with breast cancer progression [21, 22].

In our study, we investigated the role of C-phycocyanin as an anti-breast cancer agent on triple-negative breast cancer MDA-MB-231 cells in vitro and uncovered the molecular mechanism of anti-cancer activity. We found that C-phycocyanin effectively inhibited MDA-MB-231 cell proliferation, induced cell apoptotic and triggered G0/G1 cell cycle arrest. Furthermore, the molecular mechanism of cell cycle arrest caused by C-phycocyanin might be attributed to down-regulate the expression of Cyclin D1 and CDK2, and at the same time up-regulate the protein expression levels of p21 and p27 in MDA-MB-231 cells. Moreover, we uncovered that C-phycocyanin-mediated apoptosis was regulated by the inhibition of the ERK pathway and the activation of the JNK pathway and p38 MAPK pathway.

## Methods

### Materials

C-Phycocyanin was extracted and purified in our lab, and dissolved in PBS as a stock solution and conserved at − 20 °C [23]. The cell cycle and apoptosis analysis kit and annexin V-FITC/PI apoptosis detection kit were purchased from Shanghai YEASEN Biotechnology Co., Ltd., Shanghai, China. The TUNEL detection kit was obtained from Beyotime Biotechnology, Shanghai, China. CCK8 and all other chemicals were of analytic grade and were also purchased from Beijing Solarbio Science & Technology, Beijing, China. Mouse anti-human COX-2, Cyclin D1, Cyclin E, CDK2, CDK4, p21, p27, Fas, cleaved-caspase 3, pro-caspase 3, ERK1/2, p-ERK1/2, JNK, p-JNK, p38 MAPK, p-p38 MAPK, AKT, and p-AKT monoclonal antibodies were obtained from Santa Cruz Biotechnology (Santa Cruz, CA, USA). Antibodies against β-actin and all the second antibodies were purchased from Sigma-Aldrich.

### Cell culture

Human breast cancer cell line MDA-MB-231 was obtained from the Cell Bank of Chinese Academy of Sciences (Shanghai). MDA-MB-231 was cultured in high glucose DMEM supplemented with 10% (v/v) FBS, 100 mg/ml streptomycin and 100 units/ml penicillin in a humidified incubator with 5% CO_2_/95% air atmosphere at 37 °C.

### Cell viability assay

The effect of C-phycocyanin on MDA-MB-231 cell was detected using CCK8 assays. MDA-MB-231 cells (1 × 10^4^ cells per well) were plated into 96-well cell culture plates for 24 h. Then the medium was replaced with fresh medium with various concentrations of C-phycocyanin (0, 50, 100, 150, 200, 250, 300 μg/ml) for 24 or 48 h. After treatment, CCK8 was added into the medium according to manufacturer’s instructions for 2 h. Finally, the absorbance value was measured at 490 nm and the absorbance value was positively correlated with cell viability.

### Clonogenic assay

MDA-MB-231 was incubated in a six-well plate at about 1000 cells per well for 24 h, and then treated with different concentrations of C-phycocyanin (0, 50, 100, 150, 200, 250 μg/ml) for another 24 h. After incubation for 10 days, cells were washed with PBS twice, fixed with methanol for 15 min, stained with 0.5% crystal violet for 15 min at room temperature, and then observed under light microscope.

### Analysis of cell cycle and apoptosis by flow cytometry

The synchronized cells treated with different concentrations of C-phycocyanin (0, 100, 200 μg/ml) were collected using 0.25% trypsin, centrifuged (800 rpm), and washed with cold PBS twice. The synchronized cells were resuspended in pre-cooling 70% ethanol at 4 °C for 4 h. The synchronized cells were incubated with propidium iodide solution (20 μg/ml PI, 0.1% Triton X-100 staining solution, 0.1 mg/ml RNase A) for 30 min. The DNA contents distribution was determined by the BD Biosciences FACSCanto II Analyzer. The number of cells per sample was at least 2 × 10^4^. The analysis of apoptosis was detected using Annexin V-FITC apoptosis detection kit according to the manufacturer’s recommendations, the MDA-MB-231 cells with or without C-phycocyanin treatment was collected using 0.25% Trypsin, centrifuged (800 rpm), and washed with cold PBS twice. Then cells were resuspended in 1× binding buffer at a density of 1 × 10^6^ cells/ml. 5 μl Annexin V-FITC and 10 μl propidium iodide (PI) were added and incubated for 30 min at room temperature in the dark. The cell apoptosis was determined by the BD Biosciences FACSCanto II Analyzer. All experiments were repeated three or more times.

### Terminal dUTP nick-end labeling (TUNEL) assay

For the detection of C-phycocyanin-treated MDA-MB-231 DNA integrity, MDA-MB-231 cells were detected with the TUNEL detection kit. The collected cells were washed with PBS, and then fixed with 4% paraformaldehyde/PBS solution (pH7.4) at room temperature for 15 min. The cells were immersed into 0.3% Triton X-100 PBS solution at room temperature for 15 min, incubated with 50 μl labeling reaction mixture (5 μl TdT Enzyme and 45 μl TUNEL fluorescent labeling buffer) in a 37 °C humidified chamber for 60 min, then washed with PBS twice. After labeling, MDA-MB-231 cells were counterstained with DAPI and visualized under a fluorescence microscopy.

### cDNA synthesis and real-time PCR

Total RNA was extracted using TRIzol reagent (Invitrogen) according to the manufacturer’s instruction. The cDNA was synthesized with 1 μg total RNA and random primers by reverse transcription. The mRNA expression levels of genes were detected by real-time quantitative reverse transcription PCR in the ABI 7900HT real-time PCR system. Human glyceraldehyde 3-phosphate dehydrogenase (GAPDH) was normalized to the endogenous reference gene. The levels of COX-2 and GAPDH mRNA were measured by the SYBR Green I assay. COX-2 was amplified by using the primers with the sequence 5′-GATACTCAGGCAGAGATGATCTACCC-3′ (forward) and 5′-AGACCAGGCACCAGACCAAAGA-3′ (reverse). The GAPDH primer was 5′-ACCCAGAAGACTGTGGATGG-3′ (forward) and 5′-CAGTGAGCTTCCCGTTCAG-3′ (reverse). The reaction was carried out under the following conditions: 95 °C for 30 s, 45 cycles at 95 °C for 5 s, and 58 °C for 30 s. Values of each group mRNA level were calculated as 2^−ΔΔCt^ levels and performed at least four times.

### Western blot analysis

The concentrations of protein samples were detected using a BCA protein assay kit (Beyotime Biotechnology, Shanghai, China). Firstly, 40 μg protein was mixed with SDS loading buffer (5×) and then boiled for 10 min. Then the protein samples were separated by 10% SDS-PAGE, transferred to PVDF membrane (Millipore), blocked with 5% nonfat dry milk in TBS-Tween 20 (0.1%, v/v) for 1 h at room temperature and then incubated with specific primary antibodies at 4 °C for more than 12 h. After washed for three times with TBS-Tween 20 (0.1%, v/v), the membrane was incubated with the appropriate horseradish peroxidase secondary antibody for 1 h. After washed with TBS-Tween 20 (0.1%, v/v) for three times, the blots were detected using an enhanced chemiluminescence (Millipore).

### Immunofluorescence analysis

MDA-MB-231 cells were treated with 100 μg/ml C-phycocyanin for 24 h. Cells were fixed with 4% paraformaldehyde in PBS for 15 min, then permeabilized with 0.5% Triton X-100 for 15 min, and blocked with 10% goat serum for 1 h. Incubation with primary rabbit anti-human β-actin (diluted 1:250) was done overnight at 4 °C. After washing, cells were exposed to FITC-conjugated goat anti-rabbit second antibody (diluted 1:500). Washed with PBS, the nuclei were counterstained with DAPI for 7 min before imaging. Immunofluorescence analysis was detected by ImageXpress^®^ Micro, Molecular Devices, USA.

### Scratch assay

MDA-MB-231 cells (5 × 10^5^ cells per well) were plated into 6-well cell culture plates for 24 h. Scratches were created using micropipette tips, then replaced the culture medium with or without C-phycocyanin. Cells were incubated for 24 h. The scratch closure was scanned by phase-contrast microscopy and captured at the 0 and 24 h. The percentage of migration was calculated according to the formulae:

$${\text{Migration}}\left( \% \right) = {\text{Width of the scratch }}(0 - 2 4\;{\text{h}})/0\;{\text{h}} \; \times \; 100\% .$$

### Transwell migration assay

MDA-MB-231 cells were collected in DMEM medium without FBS with a final concentration of 10^6^ cells/ml. Then 100 μl cells were added into the chambers. The chambers were transferred to wells of 24-well plate containing 650 μl DMEM with 10% FBS. The transwell chambers were incubated for 24 h. After washing twice with PBS, chambers were fixed by 4% paraformaldehyde solution, and stained with 0.5% crystal violet for 15 min. Then the top-side of the chambers was wiped twice with a Q-tip. Each chamber was captured at a random location. Cell number was counted for each field of images and averaged for each chamber.

### Statistical analysis

For each measurement, three or four independent experiments were performed. Study results were represented as means ± the standard deviations. Statistical analyses were carried out by the Student’s t test or the one-way analysis of variance (ANOVA) using a statistical software package (SPSS, USA). P < 0.05 was considered as statistically significant. Statistical significance was also taken as *P < 0.05, and **P < 0.01.

## Results

### C-phycocyanin inhibited the proliferation of breast cancer MDA-MB-231 cells

**Fig. 1:**
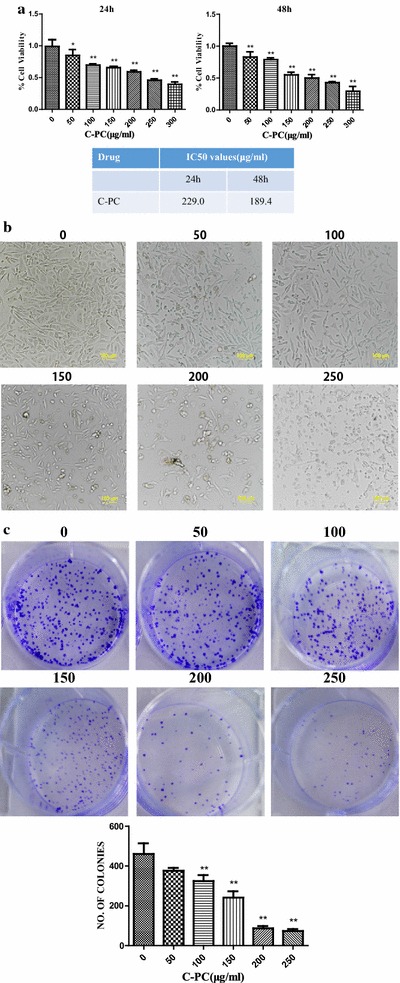
Growth inhibition effect of C-phycocyanin in MDA-MB-231 cells. **a** Cell viability of MDA-MB-231 cells is detected by CCK8 assay. IC50 values of C-phycocyanin in MDA-MB-231 cells with C-phycocyanin treatment for 24 and 48 h. Five samples were analyzed in each group, and the results are presented as the mean ± SD. **b** Morphological changes in C-phycocyanin-treated MDA-MB-231 cells. Morphological changes were observed by a microscope at 10 × 20 magnification. **c** The suppressive effect of C-phycocyanin on the colony formation of MDA-MB-231 cells

The cytotoxic effect of C-phycocyanin on breast cancer MDA-MB-231 cells was firstly evaluated by the CCK8 cell viability assay. As shown in Fig. 1a, the cell viability was reduced in a dose-dependent manner in MDA-MB-231 cell line. The IC50 value of C-phycocyanin was 229.0 μg/ml with C-phycocyanin treatment for 24 h, while the IC50 value was 189.4 μg/ml after 48 h treatment. When MDA-MB-231 cells were treated with C-phycocyanin, we observed a change in cell growth state that the cell morphology changed from long spindle-shaped to shorter spindle-shaped and flatter appearance. Furthermore, cell growth density was gradually thinning and cell growth status was significantly worse (Fig. 1b). In order to further study the inhibitory effect of C-phycocyanin on breast cancer MDA-MB-231 cells, we carried out clonogenic assay. The clonogenic assay showed that the colony formation was significant reduced when MDA-MB-231 cells were treated with C-phycocyanin, and the colony formation was reduced in a dose-dependent manner in MDA-MB-231 cell line (Fig. 1c). In view of the above results, our data suggested that C-phycocyanin could potently prevent the proliferation of breast cancer MDA-MB-231 cells.

### C-Phycocyanin induced G0/G1 cell cycle arrest in MDA-MB-231 cells

**Fig. 2:**
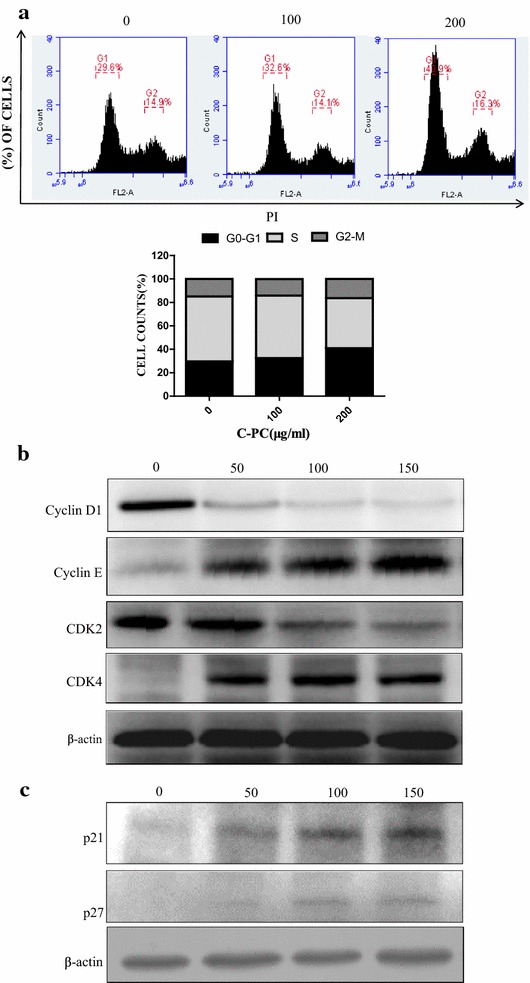
Effects of C-phycocyanin on cell cycle distribution and the expressions of cyclins, CDKs, and CDK inhibitors in MDA-MB-231 cells. **a** C-Phycocyanin induced G0/G1 cell cycle arrest in MDA-MB-231 cells. Quantitative representation of cell cycle distribution after C-phycocyanin treatment for 24 h. **b** The expressions of Cyclin D1, Cyclin E, CDK2 and CDK4 were determined by western blot. **c** The expressions of p21 and p27 were determined by western blot

Since C-phycocyanin inhibited MDA-MB-231 cells proliferation, we further explored the effects of C-phycocyanin on cell cycle progress. The synchronized cells were treated with C-phycocyanin for 24 h, and then followed by flow cytometry analysis to examine the cell cycle progress. As shown in Fig. 2a, the G1 peaks increased from 29.6 to 32.6%, and finally changed to 40.9% after treatment with 100 and 200 μg/ml C-phycocyanin, respectively. In addition, the accumulation of cells in the G1 phase was accompanied by a decrease in the population of cells in the S phase. Indeed, the S peaks decreased from 55.5 to 53.3% and then 42.8% after treatment with 100 and 200 μg/ml C-phycocyanin, respectively. Therefore, these results indicated that C-phycocyanin induced G0/G1 phase cell cycle arrest in MDA-MB-231 cells.

C-Phycocyanin treatment in MDA-MB-231 cells could be observed to have a profound effect on cell-cycle progression due to an increased population of cells in the G1 phase. To further investigate the proposed mechanism of C-phycocyanin inducing G1 phase arrest of MDA-MB-231 cells, we tested the levels of cell cycle regulatory proteins involved in G1 to S transition which included Cyclins (Cyclin D1 and Cyclin E), cyclin dependent kinases (CDK2 and CDK4) and CDKIs (CDK inhibitor p21 and p27). It has been well characterized that CDK4 is associated with d-type Cyclins while CDK2 is associated with E-type Cyclins [24]. To investigate biological pathways of G1 arrest induced by C-phycocyanin via the regulatory protein expression, MDA-MB-231 cells were synchronized and treated with C-phycocyanin for 24 h. After treatment, the protein expression levels of Cyclin D1 and CDK2 decreased in a dose-dependent manner, as shown in Fig. 2b, while the expression levels of Cyclin E increased. Meanwhile, the protein expression levels of CDK4 first increased and then decreased in C-phycocyanin-treated MDA-MB-231 cells.

Furthermore, CDKIs (CDK inhibitor p21 and p27) were often associated with the suppression of CDKs activity by forming CDK-CDKI complexes [25]. The effects of C-phycocyanin on the expression of p21 and p27 were detected by western blot, as shown in Fig. 2c. Obviously, the protein levels of p21 and p27 were upregulated in a dose-dependent manner (Fig. 2c). All these results suggested C-phycocyanin could inhibit the expressions of Cyclin D1 and CDK2, meanwhile increase p21 and p27 proteins expression in MDA-MB-231 cells.

### C-Phycocyanin induced cellular apoptosis of breast cancer MDA-MB-231 cells

**Fig. 3:**
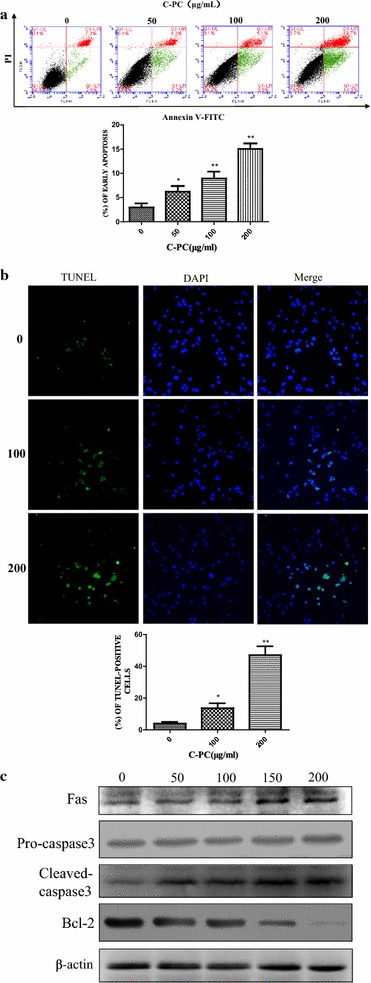
Effect of C-phycocyanin on cell apoptosis in MDA-MB-231 cells. **a** Analysis of MDA-MB-231 cells apoptosis by flow cytometry using Annexin V-FITC and PI. Quantitative representation of early apoptotic cells after C-phycocyanin treatment for 24 h. **b** Analysis of MDA-MB-231 cells apoptosis by TUNEL assay. Quantitative representation of TUNEL-positive cells after C-phycocyanin treatment for 24 h. **c** The expressions of Fas, pro-caspase 3, cleaved-caspase 3 and Bcl-2 were determined by western blot

To evaluate whether C-phycocyanin could induce apoptosis in the triple-negative breast cancer, MDA-MB-231 cells treated with C-phycocyanin were stained with Annexin V/PI, and then examined by flow cytometry. As shown in Fig. 3a, C-phycocyanin induced early apoptosis (Annexin V+/PI−) and late apoptosis (Annexin V+/PI+) in a dose-dependent manner in MDA-MB-231 cells. The percentage of early apoptotic cells increased gradually from 2.9 to 6.2, 8.6 and 14.8% with the increase of C-phycocyanin concentrations. Consistent with this result, the percentage of late apoptotic cells increased gradually from 7.3% in untreated control group to 13.7% in treated cells. In order to further study the pro-apoptosis effect of C-phycocyanin on breast cancer MDA-MB-231 cells, we carried out TUNEL assay. The TUNEL assay showed that the TUNEL-positive cells were significant increased in a dose-dependent manner when MDA-MB-231 cells were treated with C-phycocyanin (Fig. 3b). There are many studies have revealed that membrane death receptor pathways played an important role in the progress of cell apoptosis. Moreover, to further gain a deeper insight into the mechanism of apoptosis induced by C-phycocyanin, we next tested the death receptor pathway. The results of Western blot showed that C-phycocyanin upregulated the protein levels of Fas and cleaved-caspase 3 while down-regulated the protein level of Bcl-2 in MDA-MB-231 cells (Fig. 3c). Altogether, these data indicated that the C-phycocyanin could induce the MDA-MB-231 cells apoptosis.

### C-Phycocyanin inhibited the migration of breast cancer MDA-MB-231 cells

**Fig. 4:**
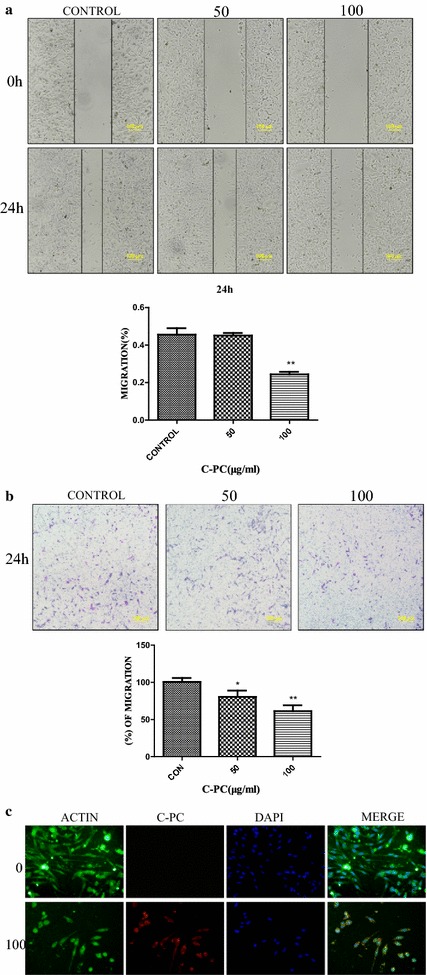
Effect of C-phycocyanin on cell migration in MDA-MB-231 cells. **a** Analysis of MDA-MB-231 cells migration by scratch assay. Quantitative representation of percentage of cell migration into the wound scratch after C-phycocyanin treatment for 24 h. Representative images of wound healing at 0 and 24 h following scratch induction and C-phycocyanin treatment. **b** Analysis of MDA-MB-231 cells migration by transwell migration assay. Quantitative representation of migration cells after C-phycocyanin treatment for 24 h. **c** Immunofluorescence analysis was performed with anti-β-actin antibodies. Morphological changes were observed in C-phycocyanin-treated MDA-MB-231 cells by a microscope at 10 × 40 magnification

Recently, some researches had found that C-phycocyanin could inhibit epithelial–mesenchymal transition (EMT) by down-regulating the expression of vimentin, type 1 collagen and fibronectin, and up-regulating the expression of E-cadherin in TGF-β1-treated cells [26]. Thus, we wanted to determine the effects of C-phycocyanin on the migration of breast cancer MDA-MB-231 cells. The wound scratch assay results showed that the migration activity of C-phycocyanin-treated MDA-MB-231 cells decreased in comparison to control cells. In wound scratch migration assays, the migration activity of C-phycocyanin-treated MDA-MB-231 cells was only 53% of the untreated control cells (Fig. 4a). Further, we verified the effect of C-phycocyanin on the metastatic activity by the transwell migration assay. Similarly, the transwell migration assays were able to show that there is an decreased the migration ability in C-phycocyanin-treated MDA-MB-231 cells (Fig. 4b). In order to further investigate whether C-phycocyanin was involved in the migration of MDA-MB-231 cells, the MDA-MB-231 cells with high ability of metastasis and mesenchymal-like phenotype were treated with C-phycocyanin. We found mesenchymal cells features were reduced while epithelial morphological features were increased in C-phycocyanin-treated MDA-MB-231 cells. For example, the morphological features of C-phycocyanin-treated cells changed from the long spindle-shaped appearance to the shorter spindle-shaped and flatter appearance by the immunofluorescence assay (Fig. 4c). Collectively these results suggested that C-phycocyanin could inhibit the migration of breast cancer MDA-MB-231 cells.

### C-Phycocyanin inhibited the COX-2 expression of breast cancer MDA-MB-231 cells

**Fig. 5:**
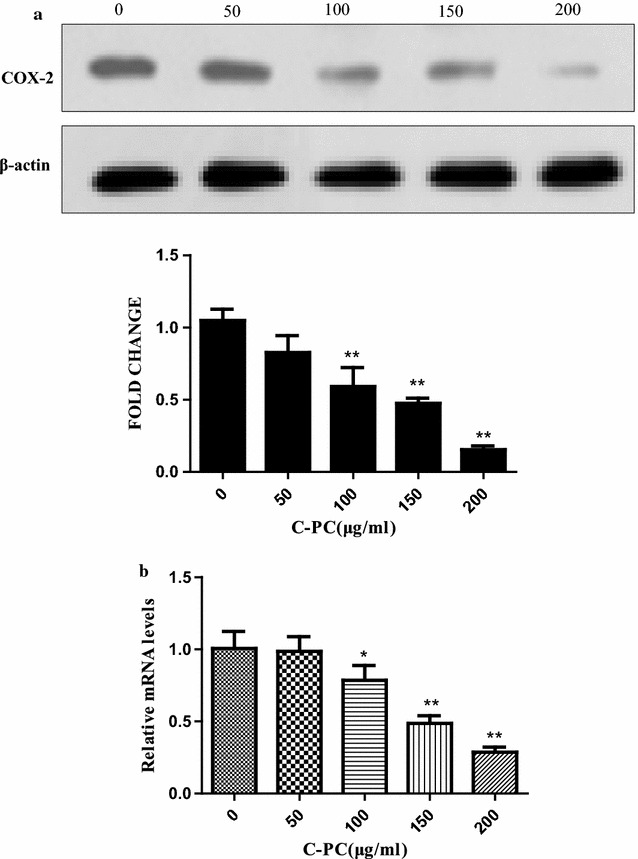
Effect of C-phycocyanin on the expression of COX-2 in MDA-MB-231 cells. **a** The expression of COX-2 was determined by western blot. The protein levels in each treatment after normalization with the levels of β-actin are shown. Data shown are representative of at least three independent experiments. **b** The expression of COX-2 was determined by qPCR. Data shown are representative of at least four independent experiments

It has been reported that COX-2 is high expression in triple-negative breast cancer and correlates with poor survival outcomes [27]. Recently, some studies have found that COX-2 is closely associated with tumor formation and progression, as well as tumor angiogenesis and metastasis [28]. It has been reported that C-phycocyanin as COX-2 inhibitor can dock with VEGF1 and inhibit colon cancer through the angiogenic pathway [29]. Thus, we determined the levels of COX-2 protein and mRNA by Western blot and qPCR, respectively. C-Phycocyanin treatment showed an effective decrease in COX-2 protein and mRNA levels in a dose-dependent manner (Fig. 5a, b).

### MAPK pathway was involved in the anti-cancer effects of C-phycocyanin on breast cancer MDA-MB-231 cells

**Fig. 6:**
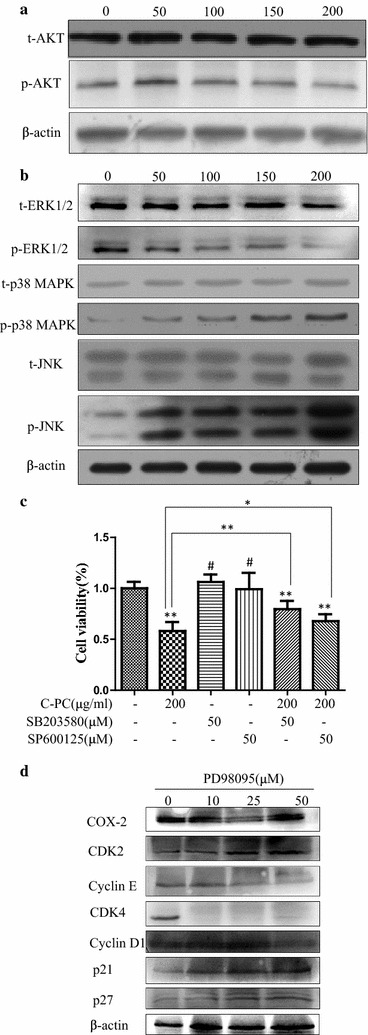
Involvement of the MAPK pathway in C-phycocyanin-induced cell death. **a** Effect of C-phycocyanin on the PI3K/AKT signaling in MDA-MB-231 cells. After treatments, the expression levels of p-AKT and total AKT were analyzed by Western blotting. **b** Effect of C-phycocyanin on the MAPK signaling in MDA-MB-231 cells. After treatments, the levels of ERK1/2, JNK and p38 MAPK and their phosphorylated forms were analyzed by Western blotting. **c** The effects of inhibitors (SB203580 or SP600125) on cell viability were evaluated by CCK-8 assay. Significant difference compared with control group are indicated as *P < 0.05, **P < 0.005, and ^#^ P > 0.05. **d** The effects of PD98059 on the levels of COX-2, CDK2, CDK4, Cyclin D1, Cyclin E, p21 and p27 were evaluated by Western blotting

The AKT and MAPK signal pathway have been reported to play crucial roles in proliferation and apoptosis progress. In particular, down-regulation expression of the ERK could prevent cancer cell proliferation, while up-regulation expression of the JNK could promote cancer cell apoptosis. In addition, it was also reported that p38 MAPK could block cell proliferation, or promote cell apoptosis. To ascertain whether the AKT and MAPK signal pathways participated in the anti-tumor effects of C-phycocyanin, so we examined the phosphorylation of MAPKs and AKT in C-phycocyanin-treated MDA-MB-231 cells. As shown in Fig. 6a, b, C-phycocyanin regulated the phosphorylation of MAPKs in a dose-dependent manner, while did not significantly alter the phosphorylation of AKT. In addition, C-phycocyanin did not significantly alter the expression levels of total AKT and MAPKs. C-Phycocyanin increased the levels of p-JNK and p-p38 and decreased the level of p-ERK in a dose-dependent manner in the MDA-MB-231 cells (Fig. 6a).

In order to further verify the role of MAPK in the anti-tumor effect of C-phycocyanin, we treated the C-phycocyanin-treated MDA-MB-231 cells with 50 μM SB203580 (p38 MAPK inhibitor) or 50 μM SP600125 (JNK inhibitor). And through CCK-8 experiment, we found the inhibition of p38 MAPK and JNK suppress the C-phycocyanin-induced cell death when MDA-MB-231 cells were treated with SB203580 and SP600125 in Fig. 6c. Then we treated MDA-MB-231 cells with 10, 25, 50 μM PD98059 (ERK1/2 inhibitor), we found the inhibition of ERK could suppress the COX-2 expression, enhances the p21 and p27 expression, and further suppress CDK4 and Cyclin E expression when MDA-MB-231 cells were treated with PD98059 in Fig. 6d. Therefore, MAPK pathway was involved in the anti-cancer effects of C-phycocyanin on breast cancer MDA-MB-231 cells.

## Discussion

Triple-negative breast cancer is a biological subtype of breast cancer, whose hallmarks is lack the expression of the breast cancer prognostic markers ER, PR and HER2 [3, 4]. Triple-negative breast cancer represents significant clinical challenge due to the abilities of highly aggressive and resistant to conventional endocrine therapy. While we currently lack targeted therapeutic strategies for triple-negative breast cancer, the discovery of novel biomarkers and selective therapies targeting these biomarkers offer potential promise for the future. So far, more researchers have found that C-phycocyanin has the anti-cancer function, which can inhibit tumor cell proliferation, induce tumor cell cycle arrest and promote tumor cell apoptosis and autophagy [6, 28]. Thus, C-phycocyanin can function as a potential anti-cancer drug. But the current study on triple-negative breast cancer has barely been reported in detail. In our study, we have confirmed that C-phycocyanin could effectively inhibit the proliferation, induce tumor G0/G1 cell cycle arrest and promote the apoptosis of the triple-negative breast cancer MDA-MB-231 cells in a dose-dependent manner. These results proved that C-phycocyanin has the anti-cancer effects on MDA-MB-231 cells.

In recent years, it has been confirmed that cell cycle dysfunction is closely interrelated with the development of tumor [30]. Therefore, cell cycle pharmacological intervention is becoming a promising approach for cancer therapy. The key to cell cycle regulation is three major cell cycle checkpoints: the G1/S checkpoints, the S phase checkpoints, and the G2/M checkpoints, which are the essential steps for the cell cycle [30]. Cell cycle checkpoints are the key to pharmacological interventions for cell cycle, which are the most effective targets for anti-cancer drugs. Pharmacological interventions further promote tumor cell apoptosis via blocking these checkpoints. Our results showed that C-phycocyanin induced the G0/G1 cell cycle arrest. After C-phycocyanin treatment, the protein expression levels of Cyclin D1 and CDK2 decreased in a dose-dependent manner in C-phycocyanin-treated MDA-MB-231 cells. Furthermore, the protein levels of CDKIs (CDK inhibitor p21 and p27) were upregulated by C-phycocyanin treatment in a dose-dependent manner. All these results suggested C-phycocyanin could inhibit the expressions of Cyclin D1, CDK2 and CDK4 and meanwhile increase p21 and p27 proteins expression in MDA-MB-231 cells.

Apoptosis is a complementary mechanism of cell proliferation. There are two major apoptotic pathways: one is the mitochondrial/cytochrome C (endogenous) pathway, in which the endogenous signal ultimately activates Caspase-9 and Caspase-3; the other pathway is the cell membrane surface death receptor (exogenous) pathway, which finally activates Caspase-8 and Caspase-3 [31]. Caspase-3 is activated in most apoptotic signaling pathways [32], and Caspase-3 will eventually induce apoptosis [33]. Thus, the key of tumor therapy is how to induce tumor cell apoptosis. We found that C-phycocyanin could up-regulate the protein levels of Fas and cleaved-caspase 3 while down-regulated the protein level of Bcl-2 in a dose dependent manner in C-phycocyanin-treated MDA-MB-231 cells. It indicated that the antitumor effect of C-phycocyanin on the MDA-MB-231 cells was mediated by induction of apoptosis.

C-Phycocyanin can function as an inhibitor of COX-2 which plays a crucial role in tumor progression and chemical resistance [34, 35]. It has been studied that inhibitors of COX-2 up-regulated E-cadherin expression in colon cancer cell lines [36]. In non-small cell lung cancer (NSCLC), the expression of COX-2 was positively correlated with tumor metastasis and invasion. The biological function of COX-2 is converting arachidonic acid to prostaglandins. Further studies found that exogenous prostaglandin E2 (PGE2) could significantly reduce E-cadherin expression in the NSCLC cells. Over-expression of COX-2 or PGE2 treatment can up-regulate ZEB1 and Snail (transcriptional inhibitors of E-cadherin) in NSCLC cells. In addition, PGE2 enhanced the binding of ZEB1 and Snail to E-cadherin at the chromatin level. Therefore, COX-2 and PGE2 are closely interrelated with tumor metastasis and invasion [37]. C-Phycocyanin has a therapeutic effect on the metastasis and invasion of tumor cells. In TGF-β1-treated MCF-7 and A549 cells, C-phycocyanin reduced the expression of fibronectin, vimentin and type 1 collagen, which increased the expression of E-cadherin. Thus, C-phycocyanin inhibited TGF-β1-induced EMT in MCF-7 and A549 cells [26]. Our results demonstrated that C-phycocyanin treatment effectively decreased in COX-2 protein and mRNA expression in a dose-dependent manner, furthermore inhibit the migration of breast cancer MDA-MB-231 cells.

As we all know the mitogen activated protein kinase (MAPK) pathway plays an important role in the development and progression of cancer [21]. When the cells encountered a variety of stimuli, such as UV irradiation, cytokines, tumor necrosis factor (TNF) and chemotherapeutic drugs and so on, JNK can be activated and function as a pro-apoptotic kinase. Some studies had documented that the JNK pathway activated caspases and regulated apoptosis-related proteins, such as Bax, Bcl-2 and p53. Further researcher had shown JNK acted on the mitochondrial death pathway. There are growing evidence indicated that the ERK pathway is in connection with the pathogenesis and progression of human breast cancer [17]. The role of p38 MAPK signaling is diverse, mainly depending on the stimulus and cell type. Sometimes, p38 MAPK had been shown to promote cell death, sometimes, while p38 MAPK enhanced cell growth and survival [19, 20]. According to our study, C-phycocyanin regulated the phosphorylation of MAPKs in a dose-dependent manner, while C-phycocyanin did not significantly alter total MAPKs. After treatment, C-phycocyanin increased the levels of p-JNK and p-p38, and decreased the level of p-ERK in a dose-dependent manner in the MDA-MB-231 cells. In a word, MAPK pathway was involved in the anti-cancer effects of C-phycocyanin on breast cancer MDA-MB-231 cells.

## Conclusions

**Fig. 7:**
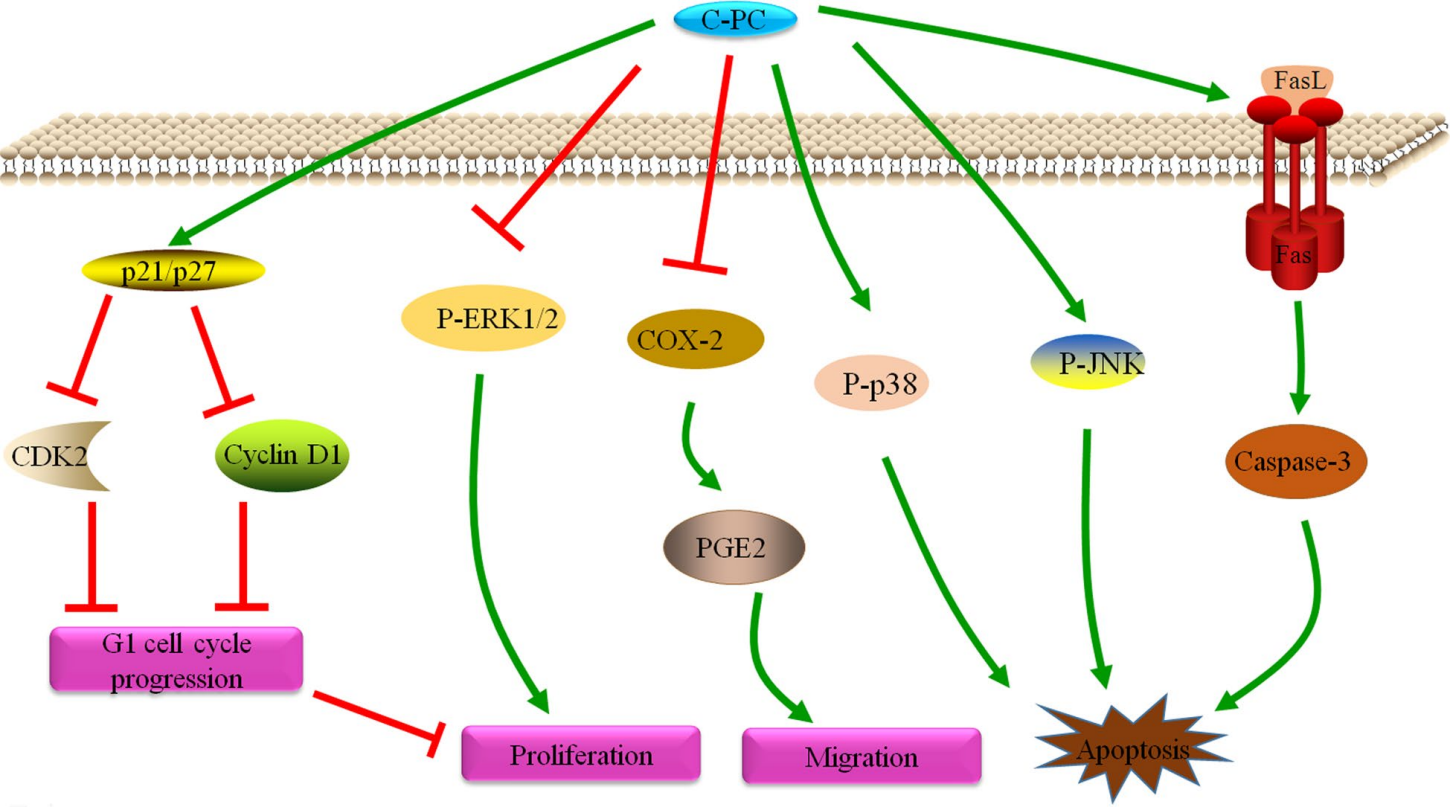
Schematic model of the proposed mechanisms for C-phycocyanin-induced apoptosis, migration and cell cycle arrest in MDA-MB-231 cells

Our study indicated that C-phycocyanin in triple-negative breast cancer MDA-MB-231 cells (i) inhibits the proliferation of tumor cell (ii) induces tumor cell cycle G0/G1 arrest (iii) promotes tumor cell apoptosis through the cell membrane surface death receptor (exogenous) pathway (iv) inhibits the COX-2 expression and tumor cell metastasis (v) down-regulates ERK signaling pathways and up-regulates JNK and p38 MAPK signaling pathways to induce tumor cell death (Fig. 7). These results proved that C-phycocyanin could serve as a promising anti-cancer therapeutic agent on triple-negative breast cancer.

## Abbreviations

C-PC: C-phycocyanin
TNBC: triple-negative breast cancer
PR: progesterone receptor
ER: estrogen receptor
MAPK: mitogen activated protein kinase
JNK: c-Jun *N*-terminal kinase
ERK: extracellular signal-regulated kinase
CDK: cyclin dependent kinase
CDKI: cyclin dependent kinase inhibitor
COX-2: cyclooxygenase-2
PGE2: prostaglandin E2
